# Laser acupuncture improving functional chronic constipation in children: a randomized controlled trial

**DOI:** 10.1007/s10103-023-03727-z

**Published:** 2023-02-15

**Authors:** Amany M. Abd El Azeem, Jehan Alsharnoubi, Marwa Abd El-Rahman Mohamed

**Affiliations:** 1 Cairo, Egypt; 2 National Institute of Laser Enhanced Sciences (N.I.L.E.S), Cairo University, Cairo, Egypt; 3 Department of Physical Therapy for Women Health, Faculty of Physical Therapy, Cairo University, Cairo, Egypt

**Keywords:** Functional chronic constipation, Laser acupuncture, Lactulose, Bristol stool scale

## Abstract

Functional chronic constipation (FCC) is a disorder caused by low fiber consumption, lack of fluid intake, lack of mobility, or side effects of medications. The objective of this study was to compare the effects of laser acupuncture and the commonly used osmotic laxative, lactulose (as the control), both combined with behavioral therapy and dietary modification, on children with FCC in a randomized controlled trial (RCT). Forty children were randomly chosen, aged 5–15 years with FCC, and randomized into two equal groups (gender ratio (50% male; 50% female), mean ± SD weight (24.2 ± 6.27 kg and 25.7 ± 7.47 kg for groups A and B, respectively)). Study group (group A): used laser acupuncture (650 nm), 30 mW, 0.15 cm^2^ spot size, 90 s per acupuncture point (ST25, ST36, ST37, BL25, and LI11). Control group (group B): lactulose syrup (1 to 3 mL/kg/day) orally, in divided doses 3 times weekly for 4 weeks, and behavioral training for both groups. Evaluations were conducted before and after the study to assess the efficacy of the therapy. Median value frequency significantly increased in groups A and B post-treatment (4 (6.75–3) and 3 (3.75–2), respectively) compared to pre-treatment (2 (2–1) and 2 (2–0.25), respectively) (*p* = 0.0001), in favor of group A (*p* = 0.01). Significant improvement of stool consistency according to Bristol stool scale (BSS) in groups A and B (*p* = 0.0001), (*p* = 0.002) respectively in favor of group A (*p* = 0.03). *T*-test, Fisher, and Wilcoxon signed rank tests were conducted to compare groups. Non-invasive, painless laser acupuncture therapy can be considered as an alternative therapy for patients with FCC.

## Introduction

Functional chronic constipation is a multifactorial disorder that is mostly related to socioeconomic status, low fiber consumption, and lack of adequate fluid intake [1]. Infrequent defecations and unintentional delay in bowel relief for 2 weeks or more associated with difficulties in bowel movement are defined as constipation [2].

There are two types of constipation (functional and organic). Functional constipation (FC) presents after the neonatal period, is not associated with any pathology, and accounts for 90% of all cases. Organic constipation accounts for the remaining 10% of cases and is characterized by the presence of pathology and presents in the neonatal period [3]. FCC diagnosis depends on examinations, symptoms, and physiological tests. Also, standard scales such as Cleveland Clinic Score, Bristol stool scale (BSS), and the Rome criteria are used [4].

Basic and advanced treatment of FC consists of lifestyle modifications and behavioral changes, medical treatment with oral or rectal laxatives, sacral nerve stimulation, biofeedback, and alternative therapy such as acupuncture, massage, and herbal therapy [5].

Acupuncture is intended to re-establish internal homeostasis through the stimulation of specific acupuncture points using laser acupuncture, acupressure, and electro-acupuncture [6]. Laser acupuncture is applied to induce bio-modulatory, stimulating, and biological changes [7].

First-line laxatives are osmotic laxatives. It promotes bowel movements by drawing water from the surrounding body tissues into the intestine. This aids in the formation of soft stool and improves bowel movement, making it easier to pass bowel movements. Some commonly used osmotic laxatives include lactulose, polyethylene glycol (PEG), and milk of magnesia [8]. Lactulose, a synthetic disaccharide fermented by bacteria within the colon, causes an increase in fecal volume and frequency of defecation and is considered to be safe for all ages [9, 10]. Osmotic laxatives are FDA-approved for the treatment of constipation. Lactulose is a laxative taken to treat difficulty pooping (constipation) and improves stool consistency and bowel movement frequency by drawing water into the bowel to make softer poo [11, 12].

One of the most important factors in constipation is diet. Constipation occurs in people of all ages as a result of a lack of fiber or fluid consumption. Fiber is the most effective constipation bowel regulator because it softens hard stool and bulks up loose stool. A well-balanced diet must include both soluble and insoluble fibers. Soluble fiber dissolves quickly in the intestines and takes on a softer, gel-like texture, aiding digestion. On the other hand, insoluble fiber passes almost unchanged through the intestines, resulting in a bulky stool [13].

Consuming fiber may reduce whole-gut transit time by increasing luminal volume and thus peristalsis. It can also have an immediate effect on bulking by causing water retention, which normalizes stool shape and improves stool volume by increasing microbial biomass and fermentation byproducts such as short-chain fatty acids. Gut transit time may be increased indirectly by lowering luminal pH, with possible consequences for gut flora [14].

Combining behavior therapy and dietary modification with frequent toileting for 5–10 min after meals, as well as a reward system, is generally beneficial. Parents should be advised to maintain a positive and encouraging attitude throughout therapy and to expect gradual improvements despite relapses [15]. It is beneficial to devote time to feces as part of toilet training. Defecation reflexes are most common in the morning and usually occur within an hour of eating. Constipated patients should sit on the toilet for 3 to 10 min once or twice daily. Make sure the child has a footrest on which they can rest their legs to effectively increase intra-abdominal pressure (Valsalva maneuver) [16].

The objective of this study was to compare the effects of laser acupuncture and the commonly used osmotic laxative, lactulose (as the control), both combined with behavioral therapy and dietary modification, on children with FCC in a randomized controlled trial (RCT).

## Patients and methods

The study was performed according to the Declaration of Helsinki principles, and approval was obtained from the ethical committee of the National Institute of Laser Enhanced Sciences (N.I.L.E.S) review board (Approval code 17/164 2021). The study was done in the Institute Pediatric outpatient clinics at Cairo University, Egypt, during the period from 2020 to 2021. The current research is RCT, including 40 children with FCC of both genders with a ratio of 50% male and 50% female in both groups.

A written informed consent was obtained from the parents of the children. They were evaluated clinically by physicians for other gastrological and neurological disorders; some variables including defecation frequency, painful defecation, hard stool, inadequate evacuation sensation, manual maneuvers, and anorectal blockage were documented. Patients with evidence of organic causes for defecation disorders, such as Hirschsprung disease, spina bifida, hypothyroidism, metabolic abnormalities, or severe liver, anal, or cardiac diseases, were excluded. The same for patients receiving any medications that can cause constipation, or having diabetes mellitus, neurologic diseases (such as seizures and cerebral palsy), also patients with previous injuries in the pelvis or spine were excluded. The patients were divided randomly into two equal groups based on the treatment line.

The participants went through a stratification process according to gender, and then, the stratified participants went through a restricted randomization process using the urn method. The patient selects one of 40 closed envelopes written in side envelopes method of treatment whether laser acupuncture or lactulose therapy.

Study group (group A):1–20 patients were treated with laser acupuncture.

Control group (group B): 21–40 patients were treated with lactulose syrup. Behavioral therapy was applied for all groups in the form of proper techniques of toileting, preferable time for defecation, as well as proper nutrition and fluid intake, and less fiber diet restriction, with a gender ratio of 50% male and 50% female in both groups.

Lactulose syrup (1 to 3 mL/kg/day) was given to group B orally in divided doses 3 times weekly for 4 weeks (we used the lower dose limit as recommended by the pediatrician).

### Laser device

**Fig. 1:**
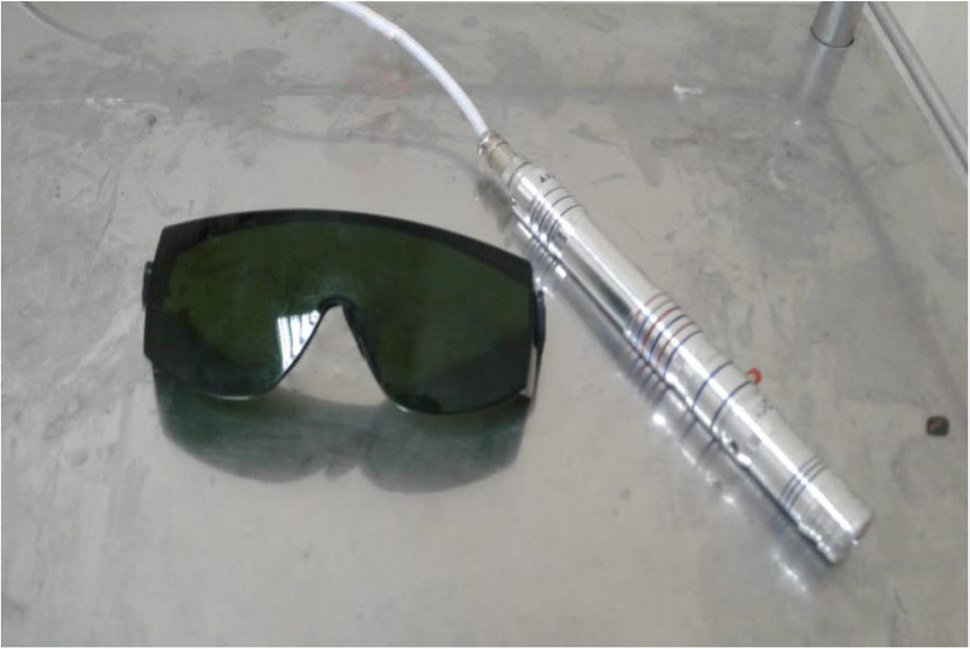
Diode laser emitter and goggles

**Fig. 2:**
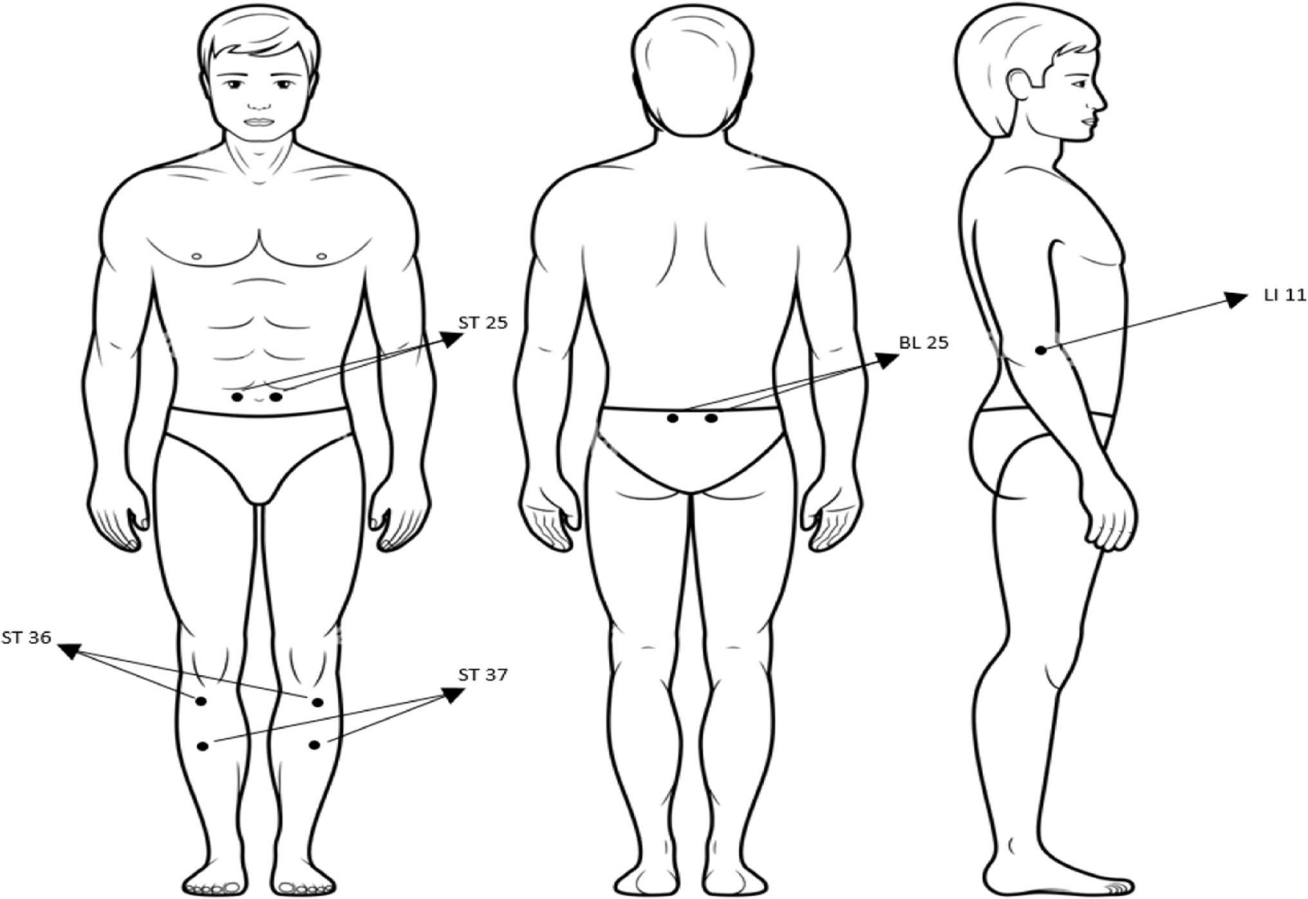
Acupuncture points

**Fig. 3:**
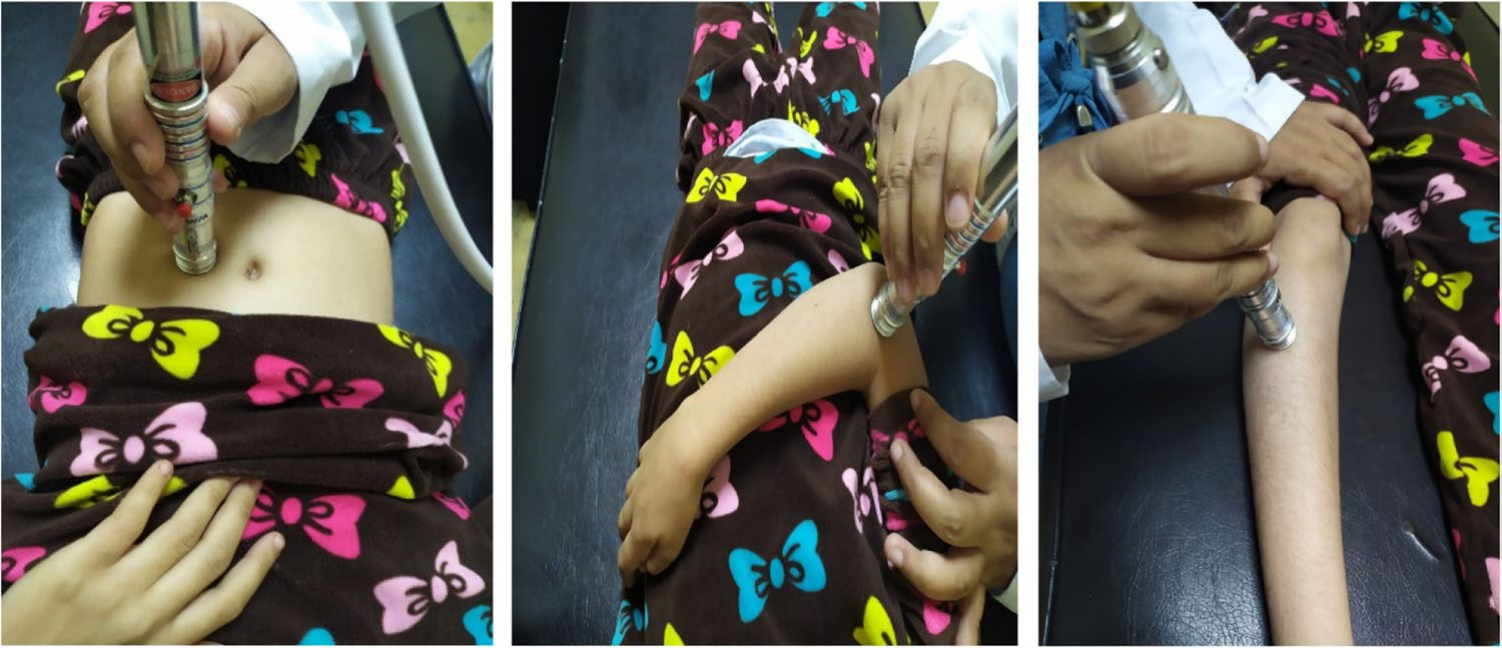
**A**, **b,** and **c** Laser acupuncture application

We used a diode laser for laser acupuncture 3 times weekly for 4 weeks using a diode laser emitter which was manufactured in our laser institute (N.I.L.E.S) in Egypt (Serial No. WM 2017), with a 650 nm wavelength, an average power of 30 mW, a 0.15 cm^2^ spot size, with continuous wave, and exposure of 90 s per acupuncture point (Fig. 1). Laser acupuncture was used at points: ST 25, ST36, and ST37; BL 25; and LI 11 (Figs. 2 and 3a, b, and c).

All people present in the examination room were wearing a special laser goggle to avoid any direct eye exposure to the laser otherwise; this is a cold laser which is not hazardous to skin.

Laser acupuncture was administered 3 times weekly for 4 weeks. The laser source has been calibrated before the intervention with both calibrated power meter (model no. 918D), manufacturer (New Port) with uncertainty ± 3%, and optical spectrum analyzer (model no. WA-1500), manufacturer (EXFO) with uncertainty ± 0.02 nm.

The participant and the operator were wearing protective eyeglasses, and no one was allowed to enter the operating room while the device was on.

Both groups received behavioral therapy advice: Sitting on the toilet for 10–15 min after breakfast and after dinner and maintain the same bathroom schedule every day, sitting in the correct position on the toilet (leaning forward while resting forearms on thighs, raising both feet on a small block (like a step stool) with feet apart, relax and lower the shoulders, and do not suppress urges to defecate and relax stomach muscles). Dietary modifications include a high-fiber diet, fruits, vegetables, whole grain, dairy, and wheat [17].

## Evaluation and follow-up

The randomization was performed by a medical assistant, while the examiner and the operator were blinded about the randomization process. The operator who delivered the intervention of laser application was under the supervision of different examiners who do not know about the study, just following the treatment regimen.

The study period was 4 months (1-month treatment and follow-up 3 months after treatment discontinuation), and the patients were evaluated by a weekly card in which the patients reported the frequency of defecations and assessment of stool consistency using the seven forms described in BSS [18], which identifies 7 types of stool; types 3 and 4 are the ideal as they are smooth, soft, and easy to pass. Patients were re-evaluated as follow-up after 3 months.

## Statistical analysis

The unpaired *t*-test was conducted for comparing the mean age, weight, and height between groups; the Mann–Whitney test was conducted for comparing the median value of frequency between groups; the Fisher exact test was conducted for comparing stool consistency distribution between groups; and Wilcoxon signed rank test was conducted for comparing the frequency and stool consistency pre- and post-treatment in each group. All statistical tests were set at *p* < 0.05 significance level (SPSS version 25 for Windows).

A sample size estimate was performed prior to the study using G*POWER statistical software (version 3.1.9.2; Germany). In order to detect a minimum difference of one in frequency between groups, approximately 40 subjects were required, with 20 subjects for each group at *α* error = 0.05, power = 80%, and effect size = 0.94.

## Results

Data obtained from both groups pre- and post-treatment and during the 3-month follow-up regarding frequency of defecation and stool consistency using the seven forms described in BSS were statistically analyzed and compared.

**Fig. 4:**
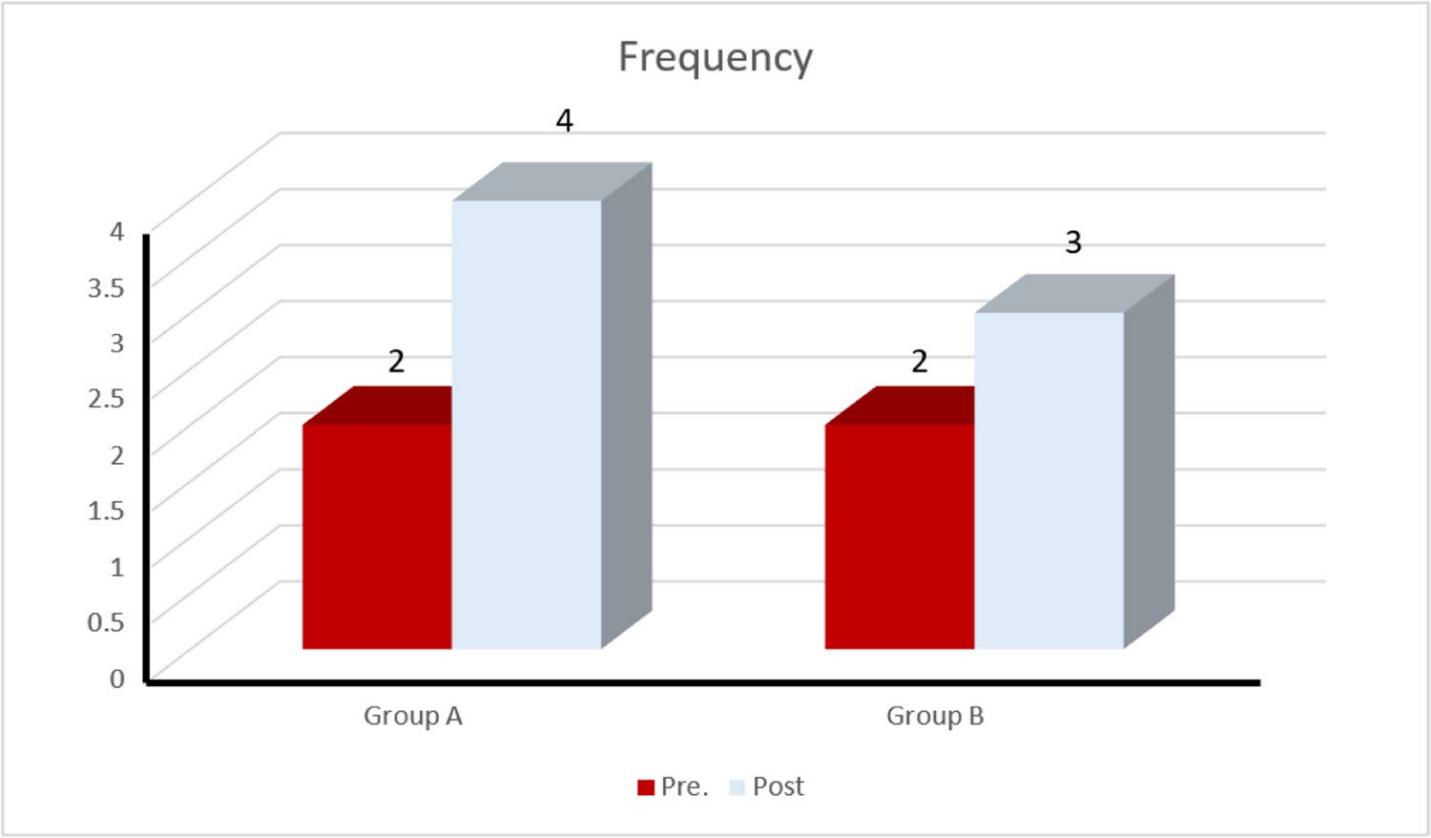
Pre-post treatment median values of frequency of the groups A (study group) and B (control group)

When comparing age, gender, weight, and height of the patient’s mean ± SD, in both groups, there was no significance. The median value of frequency before treatment for group A was 2 (2–1) and post-treatment was 4 (6.75–3) with a significant result (*p* = 0.0001); for group B, the median value of frequency before treatment was 2 (2–0.25) and post-treatment was 3 (3.75–2) with a significant result (*p* = 0.001). The median value of frequency for group A increased significantly compared to group B (*p*-value = 0.01) (Fig. 4). Also at the 3-month follow-up, there was a significant increase in the median value of frequency for group A compared with that of group B (*p*-value = 0.03), as shown in Table 1.

**Table 1:** Comparison of the median values of frequency at follow-up between groups A and B

	Group A	Group B	*U* value	*p*-value	Sig
	Median (IQR)	Median (IQR)			
Follow up frequency	4 (8–3)	3 (4.75–2)	123	0.03	S

*IQR*, interquartile range; *U* value, Mann–Whitney test value

**Fig. 5:**
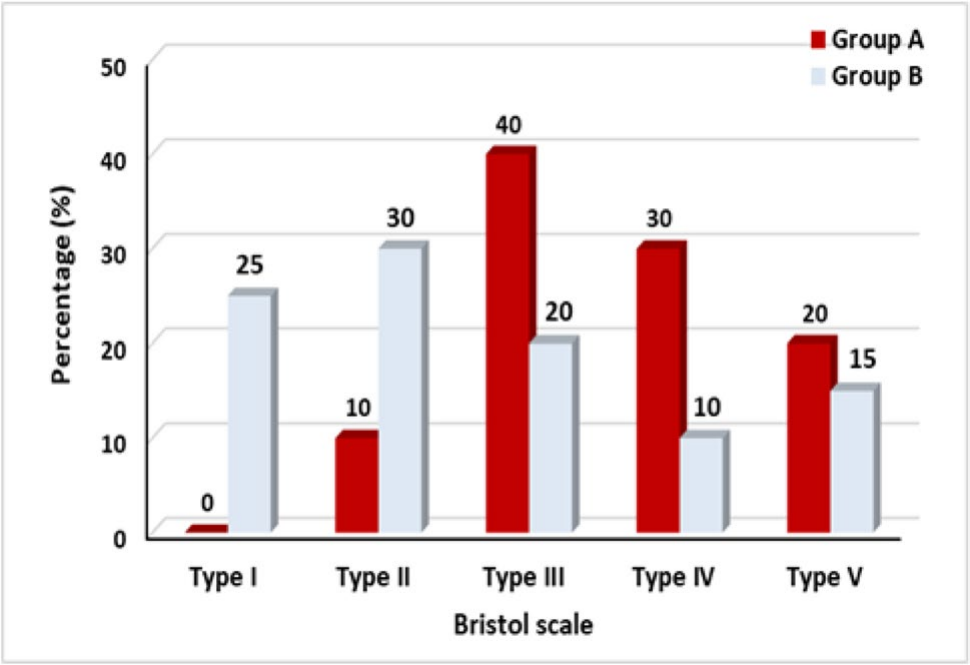
Post-treatment stool consistency of Bristol scale of groups A (study group) and B (control group)

And when comparing stool consistency distribution between groups, we found a significant improvement post-treatment in group A (*p*-value = 0.0001), as shown in Table 2. Also, there was a significant improvement post-treatment in group B (*p*-value = 0.002), as shown in Table 3, with a significant difference in favor of group A (*p*-value = 0.03) shown in Fig. 5.

**Table 2:** The stool consistency comparison between pre- and post-treatment Bristol scale distribution of group A

Bristol scale	Before	After	*Z*-value	*p*-value	Sig
Type 1	8 (40%)	0 (0%)	− 3.92	0.0001	S
Type 2	9 (45%)	2 (10%)			
Type 3	3 (15%)	8 (40%)			
Type 4	0 (0%)	6 (30%)			
Type 5	0 (0%)	4 (20%)			
Type 6	0 (0%)	0 (0%)			
Type 7	0 (0%)	0 (0%)

**Table 3:** The stool consistency comparison between pre- and post-treatment Bristol scale distribution of group B

Bristol scale	Pre	Post	*Z*-value	*p*-value	Sig
Type 1	11 (55%)	5 (25%)	− 3.07	0.002	S
Type 2	6 (30%)	6 (30%)			
Type 3	3 (15%)	4 (20%)			
Type 4	0 (0%)	2 (10%)			
Type 5	0 (0%)	3 (15%)			
Type 6	0 (0%)	0 (0%)			
Type 7	0 (0%)	0 (0%)

With the exception of group B, no major adverse effects were detected in our research, such as transient abdominal cramps for 2 patients. While group A had no adverse effects during the period of therapy.

## Discussion

FCC is a common child healthcare issue, with prevalence ranging from 0.7 to 29.6% and a female-to-male ratio of 2.1:1. FCC has varied prevalence between geographic regions. In North and South America, the ratio (including infants and adolescents) ranges between 10 and 23%, while in Europe (only including children), figures are between 0.7 and 12%. In Asia (including infants and adolescents), figures are between 0.5 and 29.6% [19].

Our findings are consistent with prior publications, yet there are some disparities in recovery rates between laser acupuncture and medical therapy, as reported in a clinical research review by Zhou. et al. (2012). The effective rate in an RCT on two hundred cases with FCC managed with auricular acupuncture for 2 sessions was 92.0% (92/100) in the auricular differentiation group, which was superior to 76.0% (76/100) in the auricular sticking group (*p* < 0.05) [20].

The symptoms of FCC improved from 13 to 8 on a 20-point scale in a study conducted by Chen et al. (2021) on 41 patients who underwent LLLT treatment using red LED light at 660 nm wavelength for 10 min, infrared LED light at 840 nm wavelength for 10 min, followed by infrared laser light at 825 nm wavelength for 20 min, eight sessions over 3 weeks. Also, Chen et al. concluded that LLLT therapy of a patient who had been unable to create spontaneous bowel motions resulted in the patient being able to have full evacuations after only eight LLLT sessions [21].

In agreement with our results, a retrospective observational study by Matsumoto-Miyazaki et al. (2019) included 25 patients with FCC who had acupuncture. The patients had two acupuncture treatments each week. After 10 weeks, defecation frequency improved from 3.0 (2.5, 3.5) to 3.5 (2.5, 4.5) times/week (*p* = 0.038). Defecation frequency improved from 2.0 (2.0, 2.5) to 2.5 (2.0, 3.5) days/week (*p* 0.001), whereas bisacodyl suppositories were used 1.5 (1.5, 2.0) times per week (*p* = 0.041) [22].

Moreover, a study in 2011 in a meta-analysis that included six RCTs comparing osmotic laxatives with placebo for treating chronic idiopathic constipation (CIC) found that, overall, osmotic laxatives were more effective in 193 (68.9%) of 280 patients allocated to placebo (RR = 0.50; 95% CI 0.39 to 0.63), while in another seven RCTs, of the 876 patients who were given laxatives, 351 (40.1%) did not react to treatment [23].

In contrary with our results, a random control trial on FCC receiving grain-shaped moxibustion and acupuncture was conducted; 100 cases were divided randomly into group (1) acupunctures at ST 25, SP 15, CV 6, CV 4, ST 36, ST 37, and SP 6 with moxibustion and group (2) acupunctures CV 6, ST 36, BL 25, and BL 20 by fifty-fifty. Reported that, the combination therapy of grain-shaped moxibustion and acupuncture is safe and effective, surpassing simple acupuncture therapy, with a total effective rate of 74.0% (37/50) in the acupuncture and moxibustion group, compared to 52.0% (26/50) in the acupuncture group (*p* < 0.05). In addition, when compared to the acupuncture and moxibustion groups, the improvement in defecation difficulty, defecation time, and abdominal discomfort in the acupuncture group was less (*p* < 0.05, *p* < 0.01) [24].

Also, in contrary with our results, Jarzebicka et al. reported that in an RCT on 102 patients, they compared the effect of two types of osmotic laxatives (polyethylene glycol vs. lactulose respectively) for FC in children using the BSS. Mean stool consistency according to the BSS was 4, although the differences were not statistically significant. Good clinical outcome was achieved in all patients which was statistically significant (*p* = 0.001) at week 4 and not statistically significant (*p* = 0.13) at week 12 [10].

Moreover, in 2018, a randomized, sham-controlled, double-blinded pilot research evaluated acupuncture’s clinical effectiveness and safety for FC using the BSS. Thirty people with FC were randomized to either the real acupuncture (RA) or sham acupuncture (SA) group, seen for 20 sessions over 4 weeks, with follow-up assessments every 2 weeks for 4 weeks after the intervention. The increase in BSS was significant in the RA group from week 7 (*p* < 0.05) [25].

In an educational review for theoretical and practical guidelines of using the laxatives for the management of FCC conducted by Bashir and Sizar (2021), the majority of laxatives are safe when taken as prescribed and in people who do not have any contraindications. For example, lactulose can cause nausea, bloating, vomiting, diarrhea, and stomach discomfort in addition to other negative effects. Another factor to consider is that osmotic drugs such as magnesium might produce metabolic abnormalities, particularly in the context of renal dysfunction. Osmotic drugs cause a rise in volume load and should be used with caution in patients with cardiac or renal disorders [26].

Moreover, Liu (2011) mentioned that abuse of laxatives is prevalent among patients who started using them for constipation once started. Misuse of laxatives causes nausea, vomiting, and alternating diarrhea with constipation. These individuals may have dehydration, electrolyte abnormalities, hyperuricemia, and hyperaldosteronism. Dehydration and hypokalemia can lead to renal failure. Diarrhea causes increased aldosterone output, which worsens hypokalemia [27].

While in 2019, a study reported that at a systematic review and meta-analysis of high-quality RCTs, acupuncture has been used for thousands of years to treat a variety of gastrointestinal problems including constipation, which is effective, safe, and inexpensive to patients. As a result, acupuncture became one of the most promising forms of alternative medicines. Acupuncture has been shown to be an effective treatment for FC across several RCTs over the last decade, increasing weekly spontaneous bowel movements, reducing constipation symptoms, and improving the quality of life in FC patients [28].

In general, our findings are consistent with most of these studies, but there are some discrepancies in the rates of recovery. Such variations might be caused by the use of different acupuncture sites, the duration of therapy, and the dose of pharmaceutical treatment. Parental guidance recommendations and behavioral training instructions were followed by parents in both groups. As a consequence, we believe that the attitude of the parents was the same in both groups and had no impact on the findings.

In conclusion, for patients with FCC, laser acupuncture may be regarded as an alternative therapy (safe, painless with low cost). However, further research involving a larger number of individuals should be conducted in order to corroborate these findings.
